# Impact of large choroidal vessels on choriocapillaris flow deficit analyses in optical coherence tomography angiography

**DOI:** 10.1371/journal.pone.0254955

**Published:** 2021-08-03

**Authors:** Valentin Hacker, Gregor Sebastian Reiter, Markus Schranz, Reinhard Told, Adrian Reumüller, Dominik Hofer, Irene Steiner, Ursula Schmidt-Erfurth, Stefan Sacu

**Affiliations:** 1 Vienna Clinical Trial Center (VTC), Department of Ophthalmology and Optometry, Medical University of Vienna, Vienna, Austria; 2 Christian Doppler Laboratory for Ophthalmic Image Analysis, Vienna Reading Center, Department of Ophthalmology and Optometry, Medical University of Vienna, Vienna, Austria; 3 Vienna Reading Center (VRC), Department of Ophthalmology and Optometry, Medical University of Vienna, Vienna, Austria; 4 Center for Medical Statistics, Informatics and Intelligent Systems, Section for Medical Statistics, Medical University of Vienna, Vienna, Austria; Massachusetts Eye & Ear Infirmary, Harvard Medical School, UNITED STATES

## Abstract

**Purpose:**

To investigate the impact of large choroidal vessels (LCV) on Choriocapillaris (CC) flow deficit (FD) analyses with swept-source optical coherence tomography angiography (SS-OCTA)

**Design:**

Prospective, cross-sectional study.

**Methods:**

Macular 6x6mm SS-OCTA scans were obtained from intermediate age-related macular degeneration (iAMD) and healthy eyes. Images were captured and processed according to most common standards and analyzed for percentage of flow-deficits (FD%) within four 1x1mm squares at the corners of each image. Choroidal thickness (CT), iris color and refraction error were considered as potential influential factors for LCV visibility. A linear mixed model and logistic regression models were calculated for statistical evaluation.

**Results:**

Sixty-nine iAMD and 49 age-matched healthy eyes were enrolled. LCV were visible in at least one sector in 52% of iAMD and 47% of healthy eyes. Within the iAMD group FD% were significantly lower in areas containing LCV (p = 0.0029). Increasing CT resulted in an odds ratio decrease of LCV (OR: 0.94, p<0.0001). Below a CT value of ≤118μm LCV could be expected with a sensitivity of 86% and a specificity of 85%.

**Conclusions:**

LCV can significantly affect CC FD analyses of SS-OCTA images. Their visibility is negatively associated with CT. The impact of LCV should be taken into account when performing CC FD assessments, especially in patients where reduced CT is to be expected and inclusion of affected areas should be considered carefully.

## Introduction

The choriocapillaris (CC) marks the capillary endpoint of the choroid and is located right below the Bruch´s Membrane (BM)/retinal pigment epithelium (RPE) complex. The choroid is subdivided into three vascular layers, the Haller layer, the Sattler layer and the CC, with vessel diameters becoming progressively smaller as they get closer to BM. The anatomical transition between choroidal vascular layers is fluent and without consensus definition at which diameter vessels are considered small, medium or large sized [1]. With vessel diameters slightly larger than that of other capillaries and fenestrations orientated towards the BM/RPE complex, the main function of the CC is nutrient supply to, and transport of metabolic products from photoreceptors, which makes it an anatomical structure of special interest as a potential starting point for various diseases [1, 2]. Histopathological studies have shown that CC dropouts are present in age-related macular degeneration (AMD), in which CC impairments coincide spatially with pathologic formations such as drusen [3]. Because of its great potential for longitudinal studies, non-invasive imaging of the CC with optical coherence tomography angiography (OCTA) is a very promising way to gain a more profound understanding of pathophysiological mechanisms. However, due to anatomical limitations such as the highly light scattering overlying RPE/BM complex, small size of CC vessels and varying layer-thickness of the CC, OCT assessments are prone to errors [4, 5]. Therefore, since the identification of some of these artifacts, compensatory methods have been published [6–10]. A compensation algorithm based on the structural information of OCT scans was developed to adjust for signal attenuation caused by the RPE/BM complex [6]. The exclusion of areas below major retinal vessel branches was preferentially chosen for shadowing artifacts caused by vessels of the superficial plexus, that currently cannot be removed entirely from OCTA scans [7–10]. While these methods can reduce the impact of artifacts caused by overlying structures on CC OCTA-analyses, the influence of large choroidal vessels (LCV), which are located below the CC, has not yet been investigated. The goal of this study was to assess the impact of LCV on CC flow deficits (FD) analyses and identify factors that can promote their visibility in OCTA CC en-face images in healthy and AMD affected eyes.

## Methods

This cross-sectional study was performed in accordance with the tenets of the Declaration of Helsinki and was approved by the Ethics Committee of the Medical University of Vienna. Written informed consent was obtained from all participants prior to enrolment.

### Inclusion and exclusion

Consecutive patients suffering from intermediate AMD (iAMD) (defined by the presence of drusen >125μm, without features of late AMD) [11] were included in this study between June 2017 and January 2020. An age matched healthy cohort served as a control group. The exclusion criteria comprised ocular opacities, which could have an effect on the OCTA signal, a refractive error larger than ± 6.0 diopters of spherical equivalent, history of retinal diseases other than iAMD and previous history of surgical ocular procedures other than uncomplicated cataract surgery. Diagnosis of iAMD was based on the presence of drusen larger than 125μm with or without pigmentary alterations in slit lamp fundus examination and OCT scans [11]. Eyes with macular neovascularization, or complete RPE and outer retinal atrophy [12] were excluded from the study. Only one eye was included per subject. By default, the right eye of each subject was chosen for analysis unless it did not meet the inclusion criteria.

### Optical coherence tomography angiography

6x6mm swept-source optical coherence tomography angiography (SS-OCTA) images with a resolution of 1024x1024 pixels, centered on the fovea were acquired using a Zeiss Plex Elite 9000 (Carl Zeiss Meditec Inc., Dublin, CA, USA). This instrument uses a 100-kHz light source with a central wavelength of 1060nm, a full-width at half maximum axial resolution of ~5μm in tissue, a lateral resolution at the retinal surface estimated at ~14μm and an A-scan depth of 3.0mm in tissue. Each 6x6mm scan consists of 500 twice repeated A-scans per B-scan. To minimize subject motion artifacts, FastTrac^TM^ motion correction was used during imaging.

### Image processing and analysis

**Fig 1:**
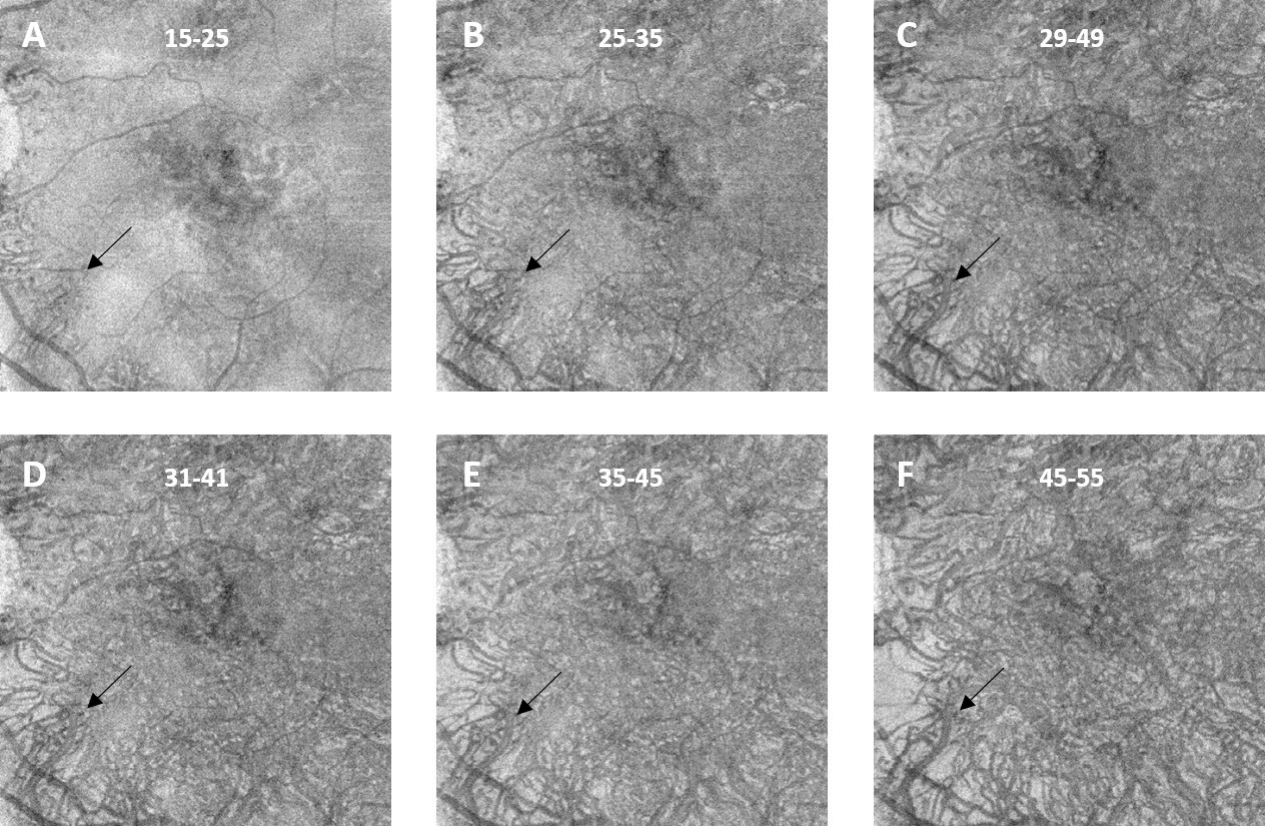
Structural en-face optical coherence tomography angiography images of an intermediate age-related macular degeneration eye, showing large choroidal vessels (LCV) within the choriocapillaris layer at different slab depths. The instrument´s automated retinal pigment epithelium fitted “RPE-fit” segmentation reference was used as a starting point from which the various offsets were applied. Manual correction was performed when necessary. LCV are first visible in the bottom left half of (A) and remain present throughout all other slab settings (black arrow) and become increasingly prominent with declining slab depth until they cover most of the image in (F). (A, B, E, F) show 10μm thick slabs with declining depth settings in 10μm increments. (C) shows the device´s default CC depth setting of 29–49μm, (D) shows the commonly used setting of a 10μm thick slab starting 31μm below the RPE-fit reference.

Image acquisition and post-processing parameters were based on most commonly used standards in literature to obtain comparable results [5, 6, 9, 13]. CC images were generated using custom slabs based on the RPE-fit segmentation of the OCTA device. All scans were checked for correct segmentation and manually corrected if necessary. Two CC slabs were exported to cover a wide range of settings used in previous publications which range from 10 to 30μm in thickness with varying distance to the BM/RPE complex [4, 7, 9, 14, 15]. A 10μm thick slab starting 31μm below the RPE-fit line as previously described [9] and a 20μm thick slab starting 29μm below the RPE-fit line, based on the standard CC segmentation settings of the device (Fig 1).

**Fig 2:**
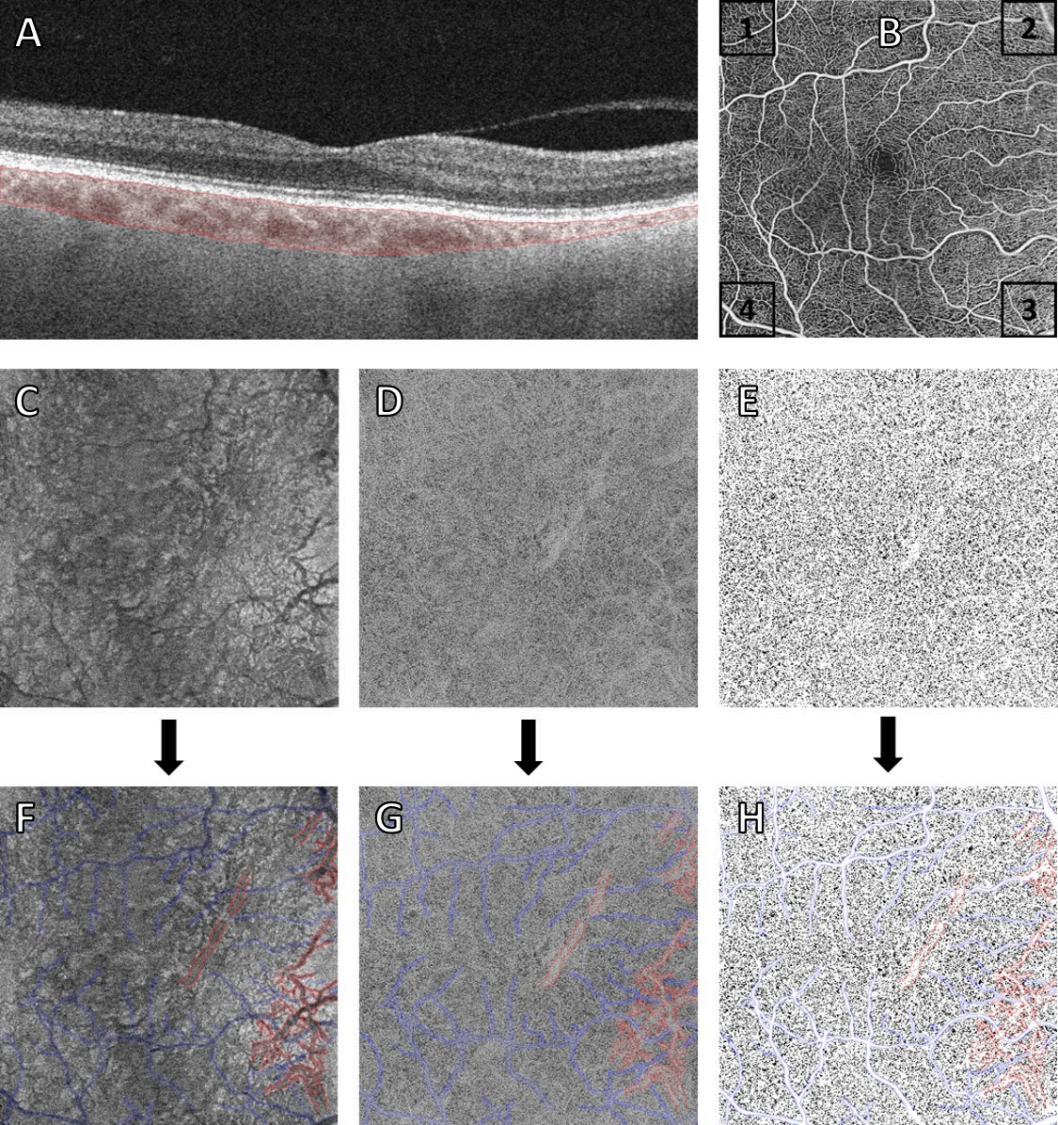
Visualization of choroidal thickness (CT) and en-face choriocapillaris (CC) images throughout different processing steps in 6x6mm scans of a healthy right eye. (A) CT (marked in red) in a B-scan showing a thickness decrease towards the nasal half of the image. (B) Layout of the four squares in a right eye. (C-E) en-face images of the CC-slab, showing the structural information scan (C), the signal compensated CC scan (D) and the binarized version of the signal compensated CC scan (E). The bottom set of images (F,G,H) shows the projection of the superficial plexus, marked in blue and large choroidal vessels (LCV) marked in red on the images above. Additionally, in the last image (H) the superficial plexus was masked for better visualization.

To compensate for image artifacts arising from light attenuation, a signal compensation method using structural information from the same slab, was performed [6]. Images were binarized with Fiji (an expanded version of ImageJ version 1.52p, available at fiji.sc, free of charge) using the Phansalkar auto local threshold method [16]. The default 15-pixel radius which is most commonly used throughout literature, as well as a 4-pixel radius adjusted to the image size as previously recommended were applied [5, 9, 17]. Areas below major branches of the retinal superficial plexus were masked and excluded from the analyses to eliminate potential projection artifacts (Fig 2) [7–10]. Instead of analyzing CC vessels which are too small to be visualized by OCTA, areas with a lack of flow information, FDs were measured and assessed as percentage per analyzed region as FD% [9]. FD analyses were performed with a custom script in a Python based environment, replicating Fiji´s analyze particle command. Previous studies reported an average CC intercapillary distance (ICD) of 24μm at the posterior pole and excluded FD-areas smaller than this metric from their analyses to reduce the influence of distortive factors such as system noise and normal intercapillary spacing [4, 5, 14]. This method was proven functional and reported to create more accurate and repeatable measurements of FDs [4]. Therefore, FDs with an area of adjacent pixels smaller than the average ICD of ~24μm were excluded from the analysis. FDs were analyzed within four 1x1mm squares at the corners of each scan in both compensated and uncompensated images (layout shown in Fig 2B). This approach was based on a previously described method, as it allows investigation of the CC without interference of pathologies located at the center of the posterior pole [18]. If drusen of any size were present in one of the squares, that single square was excluded from further analyses. Drusen were identified by reviewing individual b-scans of the four 1x1mm squares, LCV were identified based on morphological appearance on structural OCTA scans of the CC slab. The classification was performed in a binary way, divided in LCV present and LCV not present. In inconclusive cases, en-face the device´s predefined choroid slabs were used as visual aids. The presence of drusen and LCV was identified by two retinal experts (R.T., A.R.) for each of the four squares individually. If no consensus between the two experts was reached, a third expert (G.R.) was consulted. Choroidal thickness (CT) was measured at five points within each eye: at the sub-foveal center and at the center of each 1x1mm square. Measurements were taken between the outer border of BM and the inner border of the sclera drawing a virtual line perpendicular to the tangential of the foveal contour as previously described [19]. Additionally, refraction and eye color were noticed as potential influential factors for visibility of LCV.

### Statistical analysis

Statistical analyses were conducted with SAS (Version 9.4, SAS Institute Inc., Cary, NC, USA) and R (Version 3.6.2, R Foundation for Statistical Computing, Vienna, Austria) p-values < 0.05 were considered statistically significant. Quantitative demographic and clinical variables are reported as mean values ± standard deviation (SD) and group comparisons were done by two-sample t-tests for unequal variances. For qualitative variables, absolute frequencies are reported and group comparisons were done by Chi-squared tests.

### Influence of LCV on FD analyses

To investigate, whether LCV (1 measurement per quadrant) had an effect on FD analyses, a mixed model was calculated with FD% values used as the dependent variable. Presence of LCV, topographic orientation (temporal, nasal), group (iAMD, healthy control group) and the interactions of LCV and orientation, LCV and group, orientation and group were defined as independent variables. Patient was included as a random effect to account for the repeated measurements per eye. As the F-test of the interactions between LCV and orientation, and between orientation and group had a p-value greater than the significance level of 0.05 (results not shown), these interaction-terms were removed from the model. The comparison between the iAMD and the control group as well as the comparison between sectors containing LCV vs sectors without LCV were done with t-tests derived from the mixed model. The 10μm thick slab starting 31μm below the RPE-fit line, including signal compensation, a Phansalkar radius of 15 pixels and exclusion of FDs smaller than the average ICD of 24μm was chosen as our main setting. For all other settings, the analysis was done in an exploratory way, therefore we adjusted within each endpoint for the number of group comparisons (Bonferroni correction, 4 comparisons) but not for the number of settings.

### Variables influencing visibility of LCV

To investigate the effect of group (iAMD, healthy control group), CT and orientation (temporal, nasal) on visibility of LCV, univariate logistic regression models were calculated with patient set as a random factor. If a univariate model revealed a p-value < 0.05 the independent variable was included in a multivariable logistic regression model with LCV visibility set as the dependent variable and patient as a random factor to account for multiple measurements within the same eye. The effect of eye color, cCT and spherical equivalent on LCV visibility was analyzed by univariate logistic regression models, whereby the dependent variable was defined as “presence of LCV in at least one sector”. Due to the exploratory character of these analyses we did not adjust for multiple testing. Hence, the interpretation of the results is descriptive.

To determine a cutoff, that differentiates between perceptible LCV (LCV = 1) and imperceptible LCV (LCV = 0), a univariable logistic regression model was calculated with CT (measured in 4 areas) as independent variable. The best cut-off was selected by the maximal Youden-Index. Based on this cut-off, sensitivity and specificity values were calculated. To consider the repeated measurements per patient, the 95% confidence limits for sensitivity and specificity were calculated by adjusting the confidence limits using a ratio estimator [20]. As these analyses did not focus on FD%, squares containing drusen, that can affect the outcome of FD% analyses were not excluded.

## Results

Sixty-nine iAMD and 49 age-matched healthy eyes were enrolled, resulting in a total of 118 eyes from 118 patients. The mean age was 73±6.1 years (range, 57–83) in the iAMD group and 72±8.6 years (range, 55–86) in the healthy control group. The overall demographic and clinical characteristics of the two groups are shown in the provided Table 1. LCV were equally present in both slab settings with 100% conformity.

**Table 1:** Demographic and clinical characteristics of AMD patients and controls.

	iAMD	Controls	p-value
**Number of Patients (eyes)**	69 (69)	49 (49)	
**Age (years), mean ± SD**	73 ± 6.1	72 ± 8.6	0.49
**Sex (n)**			0.0008
**Female**	53	22	
**Male**	16	27	
**CT (μm), mean ± SD**			
**Sector 1**	194 ± 63	186 ± 67	0.53
**Sector 2**	148 ± 69	152 ± 71	0.76
**Sector 3**	121 ± 57	132 ± 66	0.35
**Sector 4**	157 ± 61	170 ± 60	0.29
**cCT (μm), mean ± SD**	197 ± 82	213 ± 80	0.27
**SE (absolute value in diopters), mean ± SD**	1.52 ± 1.13	1.74 ± 1.17	0.36
**Eye Color (n)**			0.27
**Brown**	14	17	
**Green**	19	12	
**Blue**	27	16	

iAMD = intermediate age-related macular degeneration; CT = Choroidal thickness, measured at the center of each 1x1mm square; cCT = Central choroidal thickness, measured at the sub-foveal center; SE = Spherical Equivalent

### Influence of LCV on FD analyses

**Fig 3:**
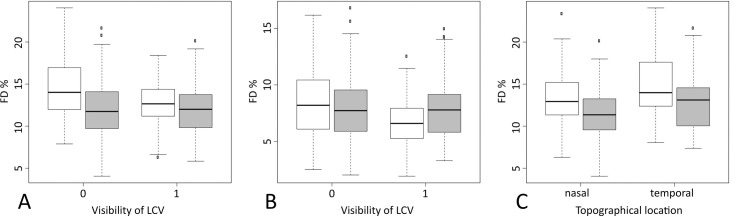
Impact of large choroidal vessels (LCV) and topographical orientation on percentage of flow deficit (FD%) values. In all three images (A-C) the intermediate age-related macular degeneration (iAMD) group is shown in white, the control group in gray. (A) Boxplots showing FD% values in sectors without (0) and with (1) visible LCV in the main setting using the standard Phansalkar radius setting of 15 pixels; (B) Boxplots showing FD% values in sectors without (0) and with (1) visible LCV in a setting using a Phansalkar radius of 4 pixels adjusted to the image size. (C) Boxplots showing FD% values in relation to their topographical location (nasal or temporal).

In our mixed model the presence of LCV, group (iAMD and control group), and the interaction between those two parameters (p = 0.0174) had a statistically significant impact on FD% analyses. If LCV were visible, mean FD% values were lower within the iAMD group (mean difference [adjusted 95% CI]: -1.33 [-2.32; -0.35], adjusted p-value = 0.0029, n = 425 observations of 118 patients (47 squares had to be excluded due to presence of drusen)). Within the control group, this difference was not statistically significant (-0.009 [-1.08; 1.06], adjusted p-value = 1) (Fig 3A). The iAMD group showed significantly higher FD% values compared to the control group, however this effect was only significant in sectors without LCV (adjusted p-value = 0.0001).

The other OCTA-slab and post-processing settings yielded similar results with exception of the 10μm thick slab starting 31μm below the RPE-fit line using a Phansalkar radius of 4, which was not able to differentiate between the iAMD and the control group, in either signal compensated, or uncompensated images. However, even with these settings, areas containing LCV yielded significantly lower FD% values within the iAMD group (Fig 3B).

Furthermore, the mixed model revealed a statistically significant effect of topographic orientation (nasal and temporal sectors) on FD% values (mean difference [95% CI] -1.16 [-1.55; -0.78], p < 0.0001), with nasal sectors showing lower FD% values across both groups (Fig 3C).

### Variables influencing visibility of LCV

**Fig 4:**
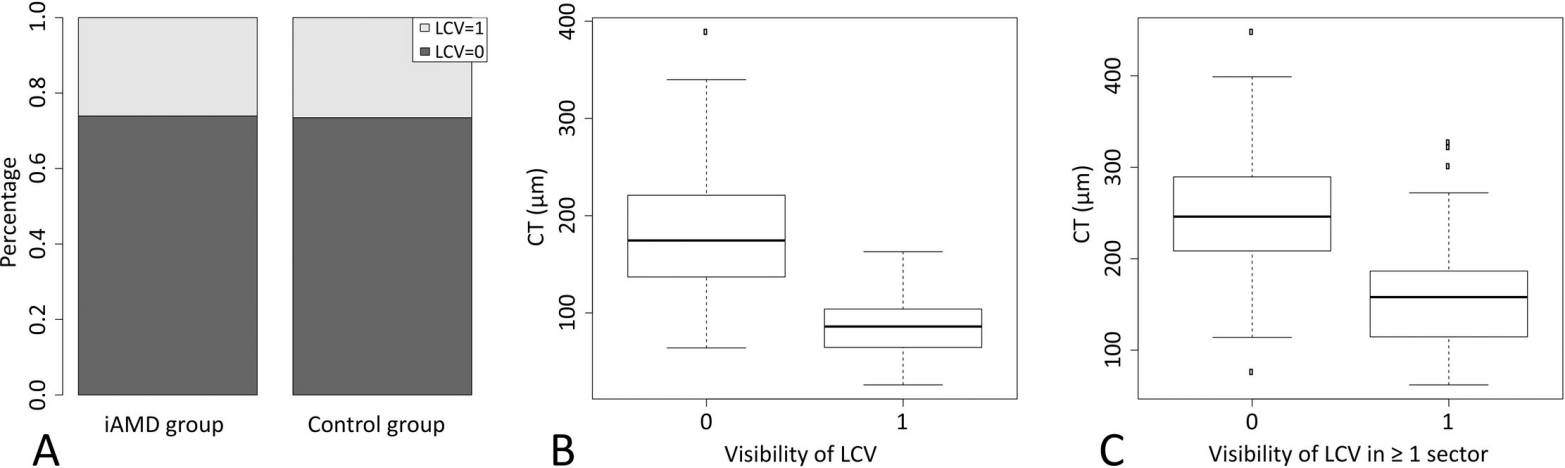
Visualization of the impact of group, choroidal thickness (CT) and central choroidal thickness (cCT) on the visibility of large choroidal vessels (LCV). (A) Barplots showing the proportions of sectors with visible LCV (shown in light gray) and without visible LCV (shown in dark gray) for the intermediate age-related macular degeneration (iAMD) and control group separately. (B) Boxplots showing the relationship between visibility of LCV and CT. (C) Boxplots showing the relationship between visibility of LCV in at least one sector and cCT.

The frequency of LCV did not differ significantly between the two groups (p = 0.99) with LCV visible in 26% and 27% of all sectors within the iAMD and control group, respectively (Fig 4A). LCV were visible in at least one sector in 52% of iAMD and 47% of healthy eyes.

With increasing CT, the odds for visibility of LCV decreased (OR [95% CI]: 0.94 [0.93; 0.96], p < 0.0001, n = 472 observations of 118 patients) (Fig 4B). In nasal areas, the odds for visibility of LCV were 6.23 times higher than in temporal areas (95% CI for odds ratio: [3.62; 10.72], p < 0.0001, n = 472 observations of 118 patients).

Calculating a multivariable logistic regression model, the interaction between the effects CT and orientation was also statistically significant (p = 0.0343). In temporal areas, the OR for CT was 0.958 [0.943; 0.974]. In nasal areas, the OR for CT was 0.931 [0.911; 0.951] which indicates that the effect of CT was greater within nasal areas compared to temporal areas.

The effect of central choroidal thickness (cCT) on presence of LCV in at least one sector was statistically significant (p < 0.0001, n = 118). With increasing cCT, the odds for visibility of LCV decreased (OR [95% CI]: 0.982 [0.976; 0.989]) (Fig 4C).

Eye color (p = 0.73, n = 105) and spherical equivalent (p = 0.1, n = 93) did not affect visibility of LCV.

The CT cutoff analysis showed that below a CT value of ≤118μm LCV could be expected with a sensitivity of 86% [81%; 92%] and a specificity of 85% [81%; 90%].

## Discussion

In this prospective cross-sectional study, the effect of LCV on CC FD assessments was investigated. FD% values were significantly lower in areas containing LCV in the iAMD group, resulting in seemingly ‘healthier’ sectors. LCV were visible in at least one sector in 52% and 47% of iAMD and healthy eyes respectively. A negative association between the visibility of LCV and CT was found in all groups.

As the lateral resolution of current OCTA devices of approximately 14μm is insufficient to resolve flow information of individual capillaries within the CC, presently used strategies inverted the analyzing process and focused on quantification of areas with decreased flow signals rather than individual vessels, so called FDs [9]. However, this approach leads to the problem that LCV–when present within the en-face projection of the CC slab, can mask FDs, resulting in seemingly healthier areas with less quantifiable FDs. In this study this effect was only observable in eyes affected by iAMD, a disease where CC flow impairments have been previously reported [7]. In healthy eyes LCV did not have a significant impact on FD analyses even though they were equally present. This finding might be attributed to the fact that the masking effect of LCV becomes most apparent in cases with an increased number of FDs. Therefore, within the healthy control group that presented significantly lower FD% values compared to the iAMD group the masking effect did not alter the results as much.

When analyzing the CC, a number of factors has to be taken into consideration to obtain reliable results, ranging from anatomical properties of the layer itself like CC- and choroidal thickness, close proximity to the highly light scattering RPE/BM complex, or imaging and post-processing settings that can influence the results [5, 6, 13, 17, 21].

The CC has a thickness of approximately 10μm [22], yet researchers used a variety of different slab settings ranging from 10 to 30μm in thickness with varying distance to BM [4, 7, 9, 14, 15]. To cover a wide range of previously described configurations, two slab settings, differing in thickness and depth, were tested in this study. The RPE-fit segmentation used to define these slabs is obtained by fitting a polynomial surface to the segmentation of the RPE. Although this has not been studied carefully, the properties of such fitting at the edges of an image can be different from the center, resulting in poorly placed CC slabs, especially if image quality is poor. To compensate for this kind of error segmentations were checked carefully and adjusted manually if necessary. With regards to LCV we noticed, that if LCV were clearly present in one slab setting, they were equally visible in the other one and even remained visible throughout a wide range of slab positions regardless of their depth and thickness as demonstrated in Fig 1.

To compensate for light attenuation caused by the RPE/BM-complex a signal compensation algorithm based on structural information of OCT scans was developed [6]. To test if the difference in FD% between regions with and without LCV was caused by light attenuation, images were analyzed in both compensated and uncompensated stages. If the presence of LCV was caused by shadowing, the signal compensation operation should hypothetically affect the results. However, the analysis of both image sets resulted in the same findings with no change in the outcome of this study.

CT is negatively correlated with age [23] and varies topographically within the human eye, with the thickest portion below the fovea and a steep negative incline towards the nasal and inferior regions [24–26]. As CT was suspected to be one of the main promotors of LCV visibility and CT does not differ significantly between healthy controls and iAMD patients [27], we included eyes from patients suffering from iAMD as well as an age matched healthy cohort for comparison. Topographical differences of CT were also found in our study population with decreased measurements in nasal regions compared to temporal regions, corresponding to previously reported results [24, 26, 28]. We found that Visibility of LCV is negatively associated with CT. The thinner the measured choroid, the higher the odds for presence of visible LCV within the CC slab become. If CT dropped below the threshold of 118μm LCV were very likely to be visible within en-face CC projections. A possible explanation could be that with increasingly thinner CT measurements, separation between individual vascular layers of the choroid becomes more difficult and LCV are more likely to appear within the boundaries of the CC slab segmentation. Therefore, CC imaging of the elderly and in peripheral regions has to be done with special attention to the presence of LCV. The same applies to imaging of patients with other underlying conditions that might affect CT such as high myopia or diabetes [19]. With regard to those results and the fact that signal compensation did not affect the outcome of our analyses, it can be suspected that visibility of LCV within the CC slab is not an imaging artifact caused by shadowing, but can be attributed to anatomical properties of choroidal layers.

Due to a lack of consensus on image acquisition and post-processing operations, different strategies have been described in literature [13]. One of the most frequently used approaches to quantify FDs is performed by thresholding images with ImageJ´s auto local threshold Phansalkar method. This method uses information within a defined pixel radius to create even thresholding throughout the image allowing the use of images containing small regional variations, e.g. areas containing poor illumination [9]. The most commonly used radius setting is the preset value of 15 pixels. However, this setting might be too large for standard OCTA images and could lead to exaggerated false positive FDs, as radius size directly influences FD extent [17]. Therefore, previous studies recommended to adjust the radius to the actual image size, aiming at a value slightly larger than the average ICD, to cover both FDs and vasculature [5, 17]. This study is one of the first to attempt the application of those recommendations, resulting in a radius of 4 pixels for 6x6mm scans with a resolution of 1024x1024 pixels.

However, whereas the standard 15-pixel radius allowed for appropriate distinction between healthy and diseased subjects in this study as well as in previous studies, the adjusted 4-pixel radius resulted in a reduced difference between the two groups. This effect was most pronounced when analyzing the 10μm thick slabs starting at 31μm below the RPE-fit line where no statistically significant difference between the two groups could be found anymore when applying the 4-pixel radius even though CC density was shown to decrease with AMD progression histologically [3]. This might be attributed to the fact that smaller radius sizes result in smaller FDs and more homogenous images. Currently used tools might not be sensitive enough to pick up subtle differences between diseased and healthy eyes when using the 4-pixel radius. Therefore, at this point the 15-pixel radius might be better suited for CC analyses until a refined analysis approach is available.

The image analysis approach chosen for this paper analyzes CC FDs at the corners of each image [18]. This method allows estimation of overall macular CC health within a patient´s eye. Furthermore, it offers the possibility to exclude individual sectors if they contain LCV and might therefore present a possible transitional approach for CC analyses until a compensation method for LCV is developed.

Another approach that might offer a solution is the averaging of multiple en-face images which can improve the visualization of capillaries and change the granular pattern of single CC OCTA-scans to a distinguishable vascular meshwork [10]. This approach appears to result in images resembling the actual histological model much closer, and could be used to exclude vessels exceeding a certain diameter threshold preserving only true capillaries of the CC. Even though this might be the most accurate approach, capturing multiple images of a patient in a clinical setting can prove difficult and time consuming, especially for patients with fixation impairments by e.g. macular atrophy, and may therefore be impractical.

Although the detection of LCV in CC en-face projections was performed by two independent retinal experts, their grading was not performed based on a vessel thickness threshold in μm but on appearance only. This is partially attributed to the fact that there is no clear consensus at what point vessels within the choroid are to be considered small, large or medium sized [1]. To compensate for this bias a sector was only treated as containing LCV if complete consensus between both readers was reached. In inconclusive cases, en-face choroid slabs were used as visual guidance to assign vessels accordingly and a third retinal expert was consulted. Furthermore, we only included iAMD patients to represent a diseased group with an already known increased amount of FDs. Therefore, it cannot be guaranteed that our results are valid for all diseases with CC involvement.

This study addresses alterations that occur in CC image analyses in the presence of LCV within the CC slab, offers an explanation for their visibility and a CT-threshold at which their perceptibility is very likely. Future studies are needed to establish compensation strategies, or vessel exclusion algorithms similar to approaches used for the superficial plexus.

In conclusion, visible LCV can significantly affect the quantification of CC FD analyses of SS-OCTA images by masking possible FDs. Visibility of LCV is negatively associated with CT, making peripheral areas where CT is usually lower particularly susceptible for this disruptive element. The impact of LCV should be taken into account when performing CC FD assessments, especially in elderly patients, or patients where reduced CT is to be expected and inclusion of affected areas should be considered carefully.
